# Nutrient Profiling of Japanese Dishes: The Development of a Novel Ajinomoto Group Nutrient Profiling System

**DOI:** 10.3389/fnut.2022.912148

**Published:** 2022-07-29

**Authors:** Chie Furuta, Hiroko Jinzu, Lili Cao, Adam Drewnowski, Yuki Okabe

**Affiliations:** ^1^Institute of Food Sciences and Technologies, Ajinomoto Co., Inc., Kawasaki, Japan; ^2^Center for Public Health Nutrition, School of Public Health, University of Washington, Seattle, WA, United States

**Keywords:** dietary recommendations, food culture, Japanese dishes, nutritional value, Nutrient Profiling System

## Abstract

Government agencies and private companies have supported the development of nutrient profiling (NP) systems to facilitate the selection of nutrient-dense foods by consumers, promote nutritious food development, and limit excessive advertising of products with low nutritional value. While most NP models were developed to assess individual foods, the Ajinomoto Group Nutrient Profiling System (ANPS) was developed to assess the overall nutritional value of cooked dishes that are culturally specific to Japan. Based on the national dietary recommendations and nutritional surveys, target values were created for 13 dish categories, while considering the combinations of meal units. For the ANPS, the four evaluating elements were protein and vegetables, which should be encouraged, and sodium and saturated fatty acids, which should be limited. The ANPS algorithm for dishes was the sum of the scores of individual elements, with a maximum of 10 points per serving. The sum of scores was then multiplied by 2.5 to convert to the 100-point scale. Convergent validity was tested using the nutrient-rich food index (NRF) score of 6.3. In total, 1,089 popular Japanese dishes were evaluated using the ANPS, and the median score of ANPS was 70.0 points (interquartile range, 55–78.8), and the average score was 67.7 (standard deviation, 16.5) points. Since salt intake is a major health risk in Japan, this tool was designed to evaluate sodium content with high sensitivity, and low-salt dishes significantly improved sodium and ANPS scores compared with regular dishes. The Pearson’s correlation coefficient between the total score of NRF 6.3 and ANPS in 1,089 dishes was *r* = 0.452 (*p* < 0.0001). This newly developed ANPS could be used to evaluate culture-specific cooked dishes per serving size. It can determine the nutritional values of dishes, with a high sensitivity to sodium content, a major Japanese nutritional issue. Further research is needed to determine the accuracy and usefulness of the ANPS as a system that would lead to changes in eating behavior nationwide.

## Introduction

Nutrient profiling (NP) models involves the ranking of individual foods according to their nutrient composition (1). The NP models have found a number of applications, ranging from front-of-pack labeling, health claims, and taxation, to the regulation of marketing and advertising to children (2, 3). Food manufacturers have used NP models to assess the nutritional value of product portfolios and to guide the innovation and reformulation of product lines. Both Nestlé and Unilever use their respective NP models to develop and provide nutritious products to consumers (4, 5).

Among the first NP models used to assess the nutritional value of individual foods are the Unilever model (now Choices International) (4), United Kingdom’s FSA-Ofcom score (2), France’s SAIN and LIM models (6), and United States’ Nutrient Rich Food Index (7). After some modifications, the FSA-Ofcom score effectively became the Health Star Rating in Australia and New Zealand (8), and the Nutri-Score in France (3). The governmental NPS, together with the front-of-package labels, have shown to positively impact the correct classifying of foods according to their nutritional quality, the nutritional quality of actual food purchasing behavior and portion size choices (9, 10). Front-of-package labels may serve as an incentive for the food industry to reformulate the products with an enhanced nutritional profile (11, 12).

The many developed NP models tend to simply penalize food containing excess calories, fats, sugar, and salt, while others attempt to capture the nutrient density of foods by incorporating multiple micronutrients, vitamins, and minerals into the scoring algorithms. Although some NP models apply the same criteria across all product categories, others are category-specific and only applicable to fruits (13), beverages, or mixed carbohydrate-rich foods (14). Thus most NP models have been developed to assess the overall nutritional value of individual food items. This system is indeed a powerful tool in cultures that consume most of their diets through processed foods. However, there are still many cultures such as South and South East Asia that consume not as many processed foods compared to the western-style diets but rather consume most of their meals through homemade cooking (15). Furthermore, in the case of Japan, the major sodium sources come from seasonings, salt added during cooking, and sauces including soy sauce and miso, a total of 61.7 and 62.9% in men and women, respectively of the total sodium intake (16). In these terms, a novel NP system (NPS) is needed to evaluate the cooked dishes’ impact on public health of the population together with the current NPS for individual processed food items. Moreover this may, impact the food industry as well, by endorsing the reformulation of recipes and products such as condiments and seasonings.

The newly proposed Ajinomoto Group Nutrient Profiling System (ANPS) in this study presents several innovations. First, it was developed to assess the overall nutritional value of dishes, that is, the meal components, as opposed to individually packaged foods. There are methods used to establish the balance of nutrients within a meal and multiple meal constituents (17), but they are not specialized for meal components. Second, this newly proposed nutrient profile is based on foods and dishes actually consumed as opposed to packaged and purchased in a store. This contrasts with the Nutri-Score, which only assesses the non-reconstituted versions of packaged foods (6). Third, the ANPS model was based on portion sizes rather than 100 g or 100 kcal measures; the former method is more informative for consumers when the dish is cooked at home, and the 100 g-based NPSs struggle to evaluate foods that contain different quantities (penalizes foods that are consumed in small quantities, while it favors those that are consumed in big quantities) (18). Finally, following the World Health Organization (WHO) principle that NP models must be adapted to the nutrient needs of a specific population and to address a particular dietary or health problem (1), as a prototype, the ANPS was developed specifically for Japan. The goal of this study was to assess the relative nutritional value of dishes and meals that are characteristic of Japanese culinary culture. The present model can serve as a guide for adapting NP models to different cultures or settings (1). Context-specific dietary recommendations and guidelines are key to improving global public health.

Japan has long been ranked as a high-income country by the World Bank (19). However, its food culture is distinct from that of most Western countries and has followed a unique pattern of evolution (20). The traditional Japanese diet is built around starchy grains and vegetables, with small amounts of animal protein. The diet is low in fat and added sugars but relatively high in sodium. However, in recent decades, Japan has been facing a growing burden of non-communicable diseases owing to population aging, urbanization, and lifestyle changes (21). The diversification of the Japanese food culture and the shift toward Western food styles, with more added sugars and fats, have been associated with the emergence of health issues in Japan. Nutrient density scores for use in front-of-pack labeling in Japan are not currently being developed. This study aimed to apply the NP approach, initially developed for individual foods, to evaluate the nutritional value of traditional Japanese cooked dishes and meal components.

## Materials and Methods

### Scope and Principles of the Ajinomoto Group Nutrient Profiling System

**FIGURE 1:**
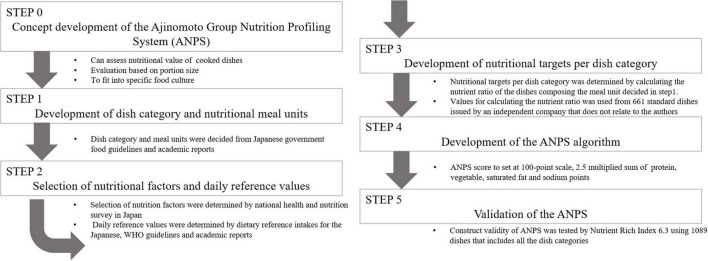
Study approach for the development of the Ajinomoto Group Nutrient Profiling System (ANPS).

This study aimed to develop a context-specific NPS that is sensitive to a particular culinary culture. The healthfulness of a single food or dietary ingredient depends on what is normally consumed. Assigning foods or dishes to common groups, subgroups, or categories is both culture-dependent and linked to habitual food patterns. Japan was chosen for the prototype model because Ajinomoto Co., Inc. is a Japanese food manufacturer, and here, the food culture is unique and diverse (20), as documented in the literature and by government-led food and nutrition surveys (22, 23). Therefore, the ANPS was designed to assess the nutritional value of cooked meals, using common servings as the basis for calculation. The development of ANPS involved the following steps: (1) development of dish categories and meal units; (2) selection of nutritional factors and daily reference values (DVs); (3) development of nutrient targets per dish category; (4) developing the ANPS algorithm; and (5) validation and testing of the ANPS as indicated in past reports (1, 24). Figure 1 shows the study approach to developing the ANPS.

### Development of Dish Categories and Meal Units

The ANPS dish categories and nutritious serving sizes were selected based on Japanese government food guidelines and previous reports (25, 26). The nutritional value of Japanese dishes (*n* = 661) were calculated using reciepes included in the Excel Eiyo-kun ver. 8 (Kenpakusha, Tokyo, Japan), and the nutrient composition of each food was based on standard tables of food composition in Japan (27). We excluded drinks, desserts, and snacks because they were not integral to meals and represented a small part of the Japanese diet (25, 28).

### Selection of Nutritional Factors and Daily Reference Values

The selection of nutritional factors and their DVs are explained in the results section.

### Development of Nutrient Targets Per Dish Category

The ideal nutrient ratio of each dish category according to each ideal meal unit was calculated from the median nutrient content data for each dish category using the recipe information provided by the nutritional calculation software (Excel Eiyo-kun ver. 8; Kenpakusha, Tokyo, Japan; Supplementary Table 1). In detail, the target nutrient value for one meal unit was set at one-third of the DV (33% DV), then allocated the median values for each dish category (Supplementary Table 1) into the seven generated meal units. As an example, in meal unit 1, the dishes are composed of a staple dish, main dish, soup, and side dish. The contribution rate of protein in the meal was 15, 52, 11, and 22% for the staple dish, main dish, soup, and side dish, respectively, using the median value of each dish indicated in Supplementary Table 1. The calculated contribution rate for all of the dishes and nutrition factors was converted into percent dietary value (%DV) and rounded up to 5% increments, to set the target values for the nutrients or food items.

### Developing the Ajinomoto Group Nutrient Profiling System Algorithm

The ANPS model generates point scores for nutrients or foods to either encourage (protein and vegetables) or limit [saturated fatty acids (SFA) and sodium] their consumption. Nutrient points for each score were based on the nutritional targets determined for each dish category. Regarding the nutrient points for the encouraged nutrients, nutrient points were set between 10 and 0, decreasing it by 10% with respect to the target values set for each dish category. Regarding the points for nutrients to be limited, points were set between 10 and 0, increasing it by 10% with respect to the target values set for each dish category. The final score was the sum of the points, and the total ANPS score was multiplied by 2.5 to convert it to a 100-point scale.

### Validation and Testing

Nutrient composition of dishes from Excel Eiyo-kun ver. 8 (Kenpakusha, Tokyo, Japan) (*n* = 661) and from our recipe website,^1^ including low salt dishes (*n* = 428), served as the principal data source for testing and validating the ANPS algorithm. The convergent validity of the ANPS was tested using the nutrient-rich food index (NRF), a well-known composite measure of nutrient density that is positively associated with the overall diet quality of the Japanese population (29–32). The variant used here, NRF 6.3, was the sum of %DVs for six encouraged nutrients minus the sum of %DVs for the three nutrients that should be limited. Serving sizes for each dish were the basis of the calculations. For the DVs of protein, SFA, and sodium, the same DVs as those of the ANPS were used. For other nutrients, the U.S. Food and Drug Administration daily values were used (33). The DVs used for the NRF calculations are listed in Supplementary Table 2.

### Statistical Analyses

The medians and interquartile ranges (IQRs) or means with standard deviations (SDs) were used to describe the nutrient values or nutrient or total points of the ANPS algorithm, as appropriate. Comparisons within dishes were performed using non-parametric Mann–Whitney U test. Pearson’s correlation coefficient was calculated to validate the ANPS compared with the NRF algorithm. All analyses were conducted using the statistical software package GraphPad Prism (GraphPad Software, CA, United States). We considered *p*-values < 0.05 to be statistically significant.

## Results

### Development of the Ajinomoto Group Nutrient Profiling System Algorithm

Step 1: Developing the dish category and meal units

The dishes were classified into four main types: staple (five subcategories), main (three subcategories), soups (three subcategories), and side (two subcategories) for a total of 13 dish subcategories (Table 1). For staple dishes, we created five subcategories, in which subcategories 1–3 were simple staple dishes of under 400 kcal. Subcategory 2 dishes contained proteins or vegetables, whereas subcategory 3 contained soup-type staples, such as udon noodles. Composite types of main and staple dishes (≥400 kcal) were separated into curry rice (subcategory 4) or soup-type ramen noodles (subcategory 5). The main dishes had three subcategories depending on whether the dish included vegetables (subcategory 7) or not (subcategory 6) or whether it was a soup (subcategory 8). For soups, there were three subcategories into which the protein and vegetable contents were classified. There were two subcategories for side dishes: subcategory 12 included nutrient-rich dishes including protein, vegetable, or energy of the dishes that did not classify as subcategory 1–11, while subcategory 13 included dishes that did not classify for subcategory 12.

**TABLE 1:** category classifications of the Ajinomoto Group Nutrient Profiling System (ANPS).

Major dish group	Subcategory number	Characteristics	Dish examples
Staple dish	1	Staple foods with simple seasonings only OR energy <400 kcal AND protein <6 g AND vegetables <50 g	Plain rice, plain bread
	2	Energy <400 kcal AND with other ingredients	Steamed rice (mixed) with red beans, hamburger
	3	Energy <400 kcal AND soup AND with other ingredients	Udon noodles with soup, soba noodles with soup
	4	Energy ≥400 kcal AND with other ingredients	Curry rice, chicken-and-egg bowl
	5	Energy ≥400 kcal AND soup AND with other ingredients	Ramen noodles
Main dish	6	Total ingredients ≥120 g AND protein ≥6 g	Grilled fish, beef steak, grilled chicken
	7	Total ingredients ≥120 g AND protein ≥6 g AND vegetables ≥50 g	Vegetable stir fry
	8	Total ingredients ≥120 g AND soup AND protein ≥6 g AND vegetables ≥50 g	Japanese hot pot
Soup	9	Soup AND protein <6 g AND vegetables <50 g	Tofu miso soup
	10	Soup AND protein <6 g AND vegetables ≥50 g	Minestrone soup
	11	Soup AND protein ≥6 g	Pork and vegetable miso soup
Side dish	12	Dishes that do not fall under category 1–11 AND (protein ≥6 g OR vegetables ≥50 g OR energy ≥100 kcal)	Boiled spinach seasoned with soy sauce
	13	Dishes that do not fall under category 1–12	Pickles

*In the ANPS system, dish category of one serving dish can be categorized by using this chart. Defining the dish category is mandatory to the evaluation of ANPS since each nutrient target values are different according to dish categories (Table 3).*

Using the 13 dish subcategories, we generated seven ideal meal units based on the “nutritious eating” project by the Japanese government (25) and our in-house registered dietitians (Table 2). The contribution ratio of each nutrient from the median values was calculated for each dish category for the seven ideal meal units. The statistics used for the calculation of the values of 661 dishes, divided into dish subcategories, are shown in Supplementary Table 1.

**TABLE 2:** Generated Japanese meal units for developing the Ajinomoto Group Nutrient Profiling System (ANPS).

Meal units		Dish subcategory number examples	Meal examples
1	Staple dish, main dish, soup, and side dish	1 + 6 + 9 + 12	White rice, grilled fish, tofu miso soup, boiled spinach seasoned with soy sauce
2	Staple dish, main dish, soup, and side dish	1 + 7 + 9 + 12	White rice, twice-cooked pork, tofu miso soup, green salad
3	Staple dish, main dish, and side dish	1 + 8 + 12	White rice, Japanese hot pot, boiled vegetables
4	Staple dish, main dish, and side dish	3 + 6 + 12	Udon noodles, tempura assorts, boiled vegetables
5	Staple dish, soup, and side dish	2 + 11 + 12	Raw egg mixed with white rice, pork and vegetable miso soup, natto
6	Staple dish and side dish	4 + 12	Curry rice, salad
7	Staple dish and side dish	5 + 12	Miso ramen, chopped vegetables

*Seven typical Japanese meal units were generated using the dish subcategories developed in Table 1. Each meal unit is described with the subcategory dish combinations with typical Japanese dish combinations.*

Step 2: Selection of nutritional factors and daily reference values

The nutrients selected for inclusion in the NP models were those usually consumed either in excess or inadequate amounts. Based on the dietary reference intake (23) and data from the national health and nutrition survey (22) for adults aged 20–60 years, we selected a number of important elements and nutrients. Inadequate intakes of energy, protein, fiber, vitamins A, D, B1, B2, B6, C, folic acid, potassium, calcium, magnesium, iron, and zinc were documented. Moreover, the Japanese population did not consume the recommended amounts of vegetables, which were once an integral component of traditional Japanese meals (20), but ate an excess of saturated fats and sodium. The prototype ANPS was designed to either encourage nutrient or food item consumption (e.g., proteins and vegetables) or to try to limit their intake (e.g., saturated fat and sodium). The DVs for each nutrient were decided as follows:

Protein: The DV for protein was set at 66 g per day and calculated from 1.1/kg/day for an average body weight of a 60-kg adult (34–38). These amounts were based on the index amino acid oxidization method (indicator amino acid oxidation technique) (39) and are higher than the current protein recommendation by the WHO, which is based on the protein maintenance requirement measured according to the nitrogen balance experiment (40). The recommended protein requirement according to the WHO method is 0.66 g/kg/day for adults. In the United States, the DVs are 0.8 g/kg/day or 56 g/day for men or 46 g/day for women (41). However, the nitrogen balance technique was thought to overestimate nitrogen intake and underestimate nitrogen excretion, leading to an overall underestimation of the requirements.

Vegetables: Global epidemiological studies have reported several benefits of high vegetable and fruit consumption, including their role in the prevention of heart disease and stroke (42–44). The WHO dietary guidelines recommend 400 g/day of vegetables and fruits (45, 46), and the Japan Ministry of Health, Labor and Welfare recommend ≥350 g/day of vegetables and ≥200 g/day of fruits during Healthy Japan 21 project (47). Since the ANPS was a system for evaluating dishes in meals, which mostly contain vegetables, we set the DV for vegetables at 350 g (25). In Japan, fruits are mostly eaten as desserts.

Saturated Fatty Acids: The WHO’s dietary guidelines are focused on reducing the intake of SFAs because of their association with cardiovascular diseases (46, 48, 49). The upper limit of the target amount of SFA was ≤10% of the total daily energy intake (50). The ANPS DV for SFAs was set at 22.2 g, based on the total daily energy intake of 2,000 kcal for adults.

Sodium: Reducing sodium intake is another priority for global public health (51, 52). The WHO dietary guidelines recommend reducing sodium to <2,000 mg/day (salt equivalent to 5 g) (53). The consumption of salt (sodium chloride) is higher in Japan than in other countries (22). Therefore, the daily Japanese target amount for sodium intake was <2.559 mg (salt equivalent to 6.5 g) in women and 2,953 mg (salt equivalent to 7.5 g) in men (23), which are above the WHO recommendations. Since the ANPS was designed to fit the food culture of the population of interest, the sodium DV was set at the mean value of the Japanese target of 2,756 mg (salt equivalent to 7 g).

Step 3: Development of nutritional targets per dish category

The contribution ratio of nutrients per dish in meal was calculated as described in materials and methods and was then converted into %DV as the target value of each dish category (Table 3). For example, for the main dish subcategory 7, the target values for protein was 20% DV (66 *g* × 20% = 13.2 *g*), vegetables 25% DV (350*g* ×25% = 87.5*g*), sodium 10% DV (2756m*g* × 10% = 275.6 mg), and SFA 15% DV (22.2 g × 15% = 3.33 *g*). We ensured that the nutrition target values of each dish category did not exceed 50% DV for the recommended nutrients and 35% DV for the nutrients which should be limited, when eaten in combination in an ideal meal unit. For example, for a meal unit 1 (the dish subcategories for staple dish, main dish, soup, and side dish were 1 + 6 + 9 + 12), the sum of the protein DV for this meal from the dish combinations was 45% (= 5 + 20 + 5 + 15), vegetable DV 30% (= 5 + 5 + 5 + 15), sodium DV 35% (= 5 + 10 + 10 + 10), and SFA DV 30% (= 5 + 15 + 5 + 5). For nutrients that should be limited, the target values were calculated so that they did not exceed approximately 100% of the total DV when the meal was eaten three times a day, and 150% DV (protein 99 g, and vegetables 525 g) for the recommended nutrients; thus ensuring a safe and tolerable upper limit for protein (54) and theoretical minimum-risk distribution of vegetables (55).

**TABLE 3:** Nutrient targets in the reference daily values DV (%) per dish category of the Ajinomoto Group Nutrient Profiling System (ANPS).

Major dish group	Subcategory number	Protein	Vegetables	Sodium	Saturated fatty acids
Staple dish	1	5	5	5	5
	2	15	5	10	10
	3	15	5	15	5
	4	25	15	25	25
	5	30	15	25	20
Main dish	6	20	5	10	15
	7	20	25	10	15
	8	30	30	20	25
Soup	9	5	5	10	5
	10	5	20	10	5
	11	15	15	10	5
Side dish	12	15	15	10	5
	13	5	5	10	5

*Values are expressed as % daily values (DV), each nutrient DV based on international, local recommendations, and scientific research.*

Step 4: Development of ANPS algorithm

**FIGURE 2:**
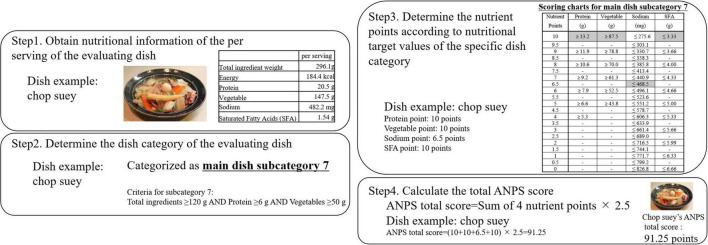
Schematic steps for the scoring algorithms of the Ajinomoto Group Nutrient Profiling System (ANPS). The scoring method and algorithm of the ANPS is determined by using the four steps indicated. The graph shows an example of the chop suey (happosai) recipe. Step 1 consists in determining the nutritional value of the dish. The recipe was obtained from the Japanese Ministry of Agriculture, Forestry and Fisheries website (https://www.maff.go.jp/j/seisan/kakou/mezamasi/recipe/recipe246.html) and the nutrient calculation was performed using Excel Eiyo-kun ver. 8; Kenpakusha, Tokyo, Japan. In step 2, the dish category is determined by using the criteria described in Table 1. In the case of chop suey, it is classified as main dish subcategory 7. In step 3, the four nutrient points are confirmed according to the target value determined from the dish category described in Table 3. In the case of chop suey, the protein value is 20.5 g per serving scoring 10 points. A serving of vegetables (147.5 g) scored 10 points, a serving of sodium (482.2 mg) scored 6.5 points, and a serving of saturated fatty acids (1.54 g) scored 10 points. Step 4 consisted in calculating the ANPS total score by adding the total nutrient points and multiplying them by 2.5. In the case of chop suey, the nutrient points add up to total of 36.5 points and the total ANPS score for chop suey is 91.25 points.

In the ANPS algorithm, for the encouraged elements, if the element of the dish exceeded the dish-specific target values, it was awarded the maximum score of 10 points. For nutrients to be limited, they were awarded a maximum of 10 points if the nutrient of the dish did not exceed the dish-specific target values. Thus, the nutrient points were decreased or increased by 10% on a 1-point scale, for the nutrients to be encouraged or limited, respectively, except for sodium. For sodium, we made 10% decrements for each 0.5 points, because there was a large divergence between the %DV and sodium amount in standard recipes eaten in Japan. In the ANPS, the values of nutrients to be limited were set backward compared with the ones to be encouraged, thus enabling the calculation of the total ANPS score as a sum. This was a rare feature compared with other score-based NPSs, such as Ofcom, Health Star Ratings, and Nutri-score. The flow diagram of the ANPS scoring algorithm is indicated in Figure 2.

### Results of the Ajinomoto Group Nutrient Profiling System Algorithm

**FIGURE 3:**
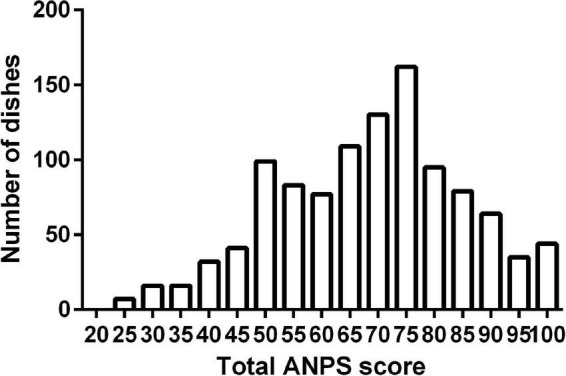
Distribution of total dishes score using the Ajinomoto Group Nutrient Profiling System (ANPS). A total of 1,089 dishes including the regular and low-salt dishes of the Ajinomoto’s recipe website and nutrition calculation software were evaluated using the algorithm indicated in Figure 2.

Using the developed ANPS algorithm, we evaluated 1,089 dishes frequently eaten in Japanese culture. The dishes included traditional (washoku) dishes and Western or international ones that are now regularly found in Japan (e.g., sandwiches, hamburgers, Chinese stir fry, and others). In addition, low-salt dishes or dishes created from low-salt seasoning products were included, to test the performance of the ANPS. The results of the ANPS score distribution are shown in Figure 3. The median of the ANPS score was 70 (IQR, 55–78.8), while the mean was 67.7 (SD 16.5) points. The ANPS points for each dish subcategory are shown in Supplementary Table 3.

In the ANPS algorithm, the total dishes evaluated ranged from 22.5 to 100 points. The highest-ranking dishes, with a score of 100 points, included mostly low-salt dishes with low SFAs and abundant in vegetables, such as eel chirashi sushi (dish subcategory 2); minced chicken, eggplant, tomato rice bowl (dish subcategory 4); Chinese-style tuna carpaccio (dish subcategory 6); steamed cabbage and pork (dish subcategory 7); pumpkin and onion sesame miso soup (dish subcategory 10); and vegetables and chicken filets with Chinese sauce (dish subcategory 12). In contrast, the lowest-scoring dishes included high-sodium and high-SFA ingredients with a low vegetable content. Some examples of the lowest-scoring dishes included garlic toast (dish subcategory 2), Sichuan dandan noodles (dish subcategory 5), pork cutlet (dish subcategory 6), vegetables with white sauce (dish subcategory 7), egg-drop soup with cheese (dish subcategory 11), and grilled potato with butter (dish subcategory 12). As listed, dishes using dairy products (milk or cheese) and high-fat animal meat, such as pork, tended to score low due to the high SFA contents. The lowest scoring dishes received points for at least protein or sodium, resulting in the lowest total score of >20 points.

The evaluation of nutritional score distribution differed within each dish subcategory (Supplementary Table 4). For most dishes, the median protein scores were high, except for side dishes 12 and 13, as most of the dishes included some sources of protein, whether the dish portion was large or small, even though they were not categorized as the main dish. For the median vegetable scores, dish subcategories 1 and 2 scored low, as these were intended as simple staple dishes that would not be eaten alone but with a main and other side dishes. For SFA, most of the dish subcategories had high median scores, with the lowest score of 5.5 in dish subcategory 11. Soups or soupy dishes in subcategories 3, 5, and 11 showed low sodium median scores, suggesting that these dishes have a big impact on sodium intake in Japanese food culture.

Using the same dataset, we extracted 303 low-salt dishes (including dishes using low-salt seasoning products: *YASASHIO* salt, *Hondashi*^®^ <low-salt>, Consomme <low-salt>, and whole chicken broth <low-salt>, sold in Japan by Ajinomoto Co., Inc., Tokyo, Japan) developed and included on our recipe website, and evaluated them in the ANPS and compared it with regular salt dishes (Table 4). The Mann–Whitney U test identified significant differences (*p* < 0.0001) in total ANPS score between regular dishes (median ANPS score, 65.0; median sodium score 4.5 points) and low-salt dishes (median ANPS score, 80.0; median sodium score, 9.0).

**TABLE 4:** Sodium and total score of the Ajinomoto Group Nutrient Profiling System (ANPS) in regular (*n* = 786) and low-salt (*n* = 303) dishes.

		Regular dish (*n* = 786)				Low-salt dish (*n* = 303)				*P*-value
				
	(*n*)	Mean	SD	Median	IQR	Mean	SD	Median	IQR	
ANPS sodium score	786	4.6	3.7	4.5	0.5–8.0	7.7	3.2	9.0	6.5–10.0	<0.0001
ANPS total score	303	63.4	15.4	65.0	52.5–75.0	79.1	13.7	80.0	72.5–88.8	<0.0001

*Values are expressed as the mean, standard deviation (SD), median, interquartile ranges (IQRs).*

*Regular and low-salt dishes were compared using the Mann–Whitney U test.*

### Validation and Testing of the Ajinomoto Group Nutrient Profiling System and Nutrient-Rich Food Index 6.3

**FIGURE 4:**
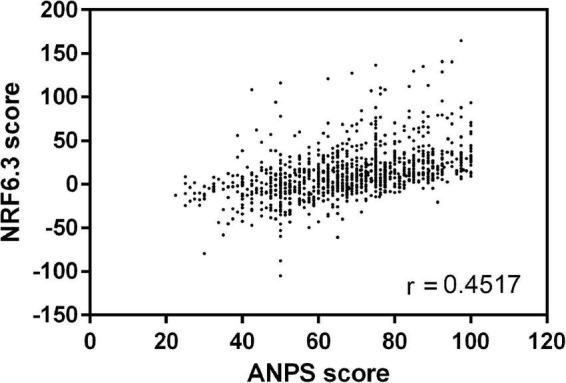
The correlation between the nutrient-rich food index (NRF) 6.3 score and the Ajinomoto Group Nutrient Profiling System (ANPS) score among 1,089 dishes.

The NRF 6.3 was used to test the convergent validity of the novel ANPS. The Pearson’s correlation coefficient between the total score of NRF 6.3 and ANPS in 1,089 dishes was *r* = 0.452 (*p* < 0.0001) (Figure 4). The correlations among the four major dish groups (staple dish, main dish, soup, and side dish) were *r* = 0.522, 0.519, 0.441, and 0.297, respectively (all *p* < 0.001). A further breakdown of the 13 subcategories is presented in Table 5. Using correlation, NRF 6.3 nutrient scores were tested against the total score of ANPS, and significant (*p* < 0.001) differences were detected for fiber, potassium, SFA, sodium, and added sugar (Supplementary Table 5).

**TABLE 5:** Pearson’s correlation coefficients among the Ajinomoto Group Nutrient Profiling System (ANPS) and nutrient-rich food index (NRF) 6.3 scores in major dish group and dish subcategories of the total 1,089 dishes evaluated.

	*r*	*P*-value
**Major dish group**		
Staple	**0.5216**	**<0.001**
Main dish	**0.5186**	**<0.001**
Soups	**0.4412**	**<0.001**
Side dish	**0.2974**	**<0.001**
**Subcategory number**		
1	**0.5886**	**<0.001**
2	**0.5957**	**<0.001**
3	**0.653**	**0.0013**
4	**0.616**	**<0.001**
5	0.2569	0.1785
6	**0.4815**	**<0.001**
7	**0.5078**	**<0.001**
8	**0.6251**	**<0.001**
9	**0.6243**	**<0.001**
10	**0.7871**	**<0.001**
11	**0.5951**	**<0.001**
12	**0.299**	**<0.001**
13	**0.506**	**0.0014**

*Significant (p < 0.05) correlations are highlighted in bold.*

## Discussion

The ANPS is a science-based system that can be used to evaluate the nutritional composition of dietary dishes that could promote nutritional diets in the Japanese population, improving public health. This is the first report of NPS that has been developed specifically for the Japanese population and designed to evaluate nationally consumed dishes.

One of the main features of the ANPS is its sensitivity to sodium. Sodium had a 0.5-point decrement, whereas the other nutrients had 1 point. During the development of the ANPS algorithm, various standards and point increments were tested for each nutrient. In the early prototype, the scoring algorithm for sodium was similar to that of SFA (1-point decrement). However, due to the high sodium content of popular dishes most scored low, resulting in a system that cannot evaluate the true dish characteristics in Japanese culture. By adapting the 0.5-point decrement of sodium, we were able to achieve high sensitivity to sodium content, a specialized feature of ANPS (Table 4). Excessive sodium intake is one of the major nutritional issues in Japan, with an average of 3,976 mg, equivalent to 10.1 g of salt (men: 4,291 mg, equivalent to 10.9 g of salt; women: 3,661 mg, equivalent to 9.3 g of salt). This value is 1.4 times higher than the %DV of 2,756 mg (22). Since nutritional evaluations, such as the NPS, must be usable, achievable, and effective, the sodium target is not yet ideal for meeting the national reference values. A gradual decrease in the ANPS standards could help to solve this, as the general population gets used to dishes with less salt. The United Kingdom has reported a successful population-level sodium reduction program, whereby the United Kingdom Food Standards Agency partnered with food manufacturers, retailers, and suppliers to implement a gradual reformulation program, successfully reducing the sodium content of bread by 17% (56). As a result, the mean consumption of dietary sodium was reduced by approximately 1 g/day over 10 years and contributed to a saving of >1.5 billion GBP per year in healthcare costs (56, 57).

The convergent validity of the system tested using the NPS NRF 6.3 showed a positive correlation with ANPS. When NRF 6.3 and ANPS were compared, staple and main dish categories showed a strong positive (*r* > 0.5) and side dish categories a weak positive (*r* = 0.297) correlation between the two (Table 5). Dishes with the strongest correlation were mostly in the main dish subcategories 6, 7, and 8. The ten most correlated dishes included fish (salmon, herring, sardines, or salmon eggs) and vegetables, most of which were low-salt, such as Chinese cabbage and sardine tsumire hot pot, grilled salmon, and salmon meuniere. For the NRF 6.3 nutrient points, these dishes scored high in vitamin D and potassium, and low in SFA and sodium, similar to the ANPS nutrient points; they scored high in all the evaluated nutrients (where higher points indicate “better” in nutrients which should be limited). The weakest correlated dishes were from the side dish subcategory (subcategory 12), which had a low NRF 6.3 score due to low nutrient density in small portions sizes (a median of 104 g ingredient weight and energy amount of 116 kcal). To further investigate the characteristics of ANPS, the correlation of individual NRF 6.3 nutrient scores was investigated. All three nutrients from nutrients to limit showed negative correlations with ANPS total scores (Supplementary Table 5). The highest correlation was in sodium (*r* = −0.324), which shows that ANPS scores are highly weighted on sodium content, a special feature of ANPS. Of the six encouraged nutrients in NRF 6.3, fiber and potassium showed weak positive correlations with ANPS, whereas other nutrients did not. Interestingly, protein, a commonly evaluated nutrient in both NRF and ANPS, showed no correlation. This can be explained from the food culture perspective. By increasing the amount of animal protein (most meat) in dishes, the total amount of SFA also increases and sodium is needed to enhance palatability. Therefore, total ANPS scores tended to be lower, even if the protein content met the nutritional target values. Fiber and potassium were correlated because the two nutrients are rich in vegetables, which is highly evaluated in ANPS.

The newly developed ANPS had many limitations, and no existing NPS is perfect. One major limitation of this system was that it depended heavily on the food culture of a region. Although the current NPS have been mostly developed in Western cultures, they can be used in many different countries and regions. However, public health issues differ within and across countries, and the WHO raised concerns regarding whether NP criteria developed for one culture, purpose, or setting could be transferred to another (1, 24). Therefore, there is a need for a tailor-made ANPS, specific for a region or country, to accommodate local food culture and nutritional issues. For example, in the present ANPS Japanese prototype, desserts and drinks were excluded as a dish category or meal unit. The main reason for this exclusion was the inability of ANPS to evaluate energy and added sugars, the primary components of snacks, desserts, and sugar-sweetened beverages. In addition, these were all considered as snacks and not as meals (25), with snacks contributing to only 8% of the daily energy ratio in Japan, compared with 14–31% in Western countries (28, 58). These should be customized in algorithms according to the food culture and issues of specific regions.

Another limitation was that the ANPS could not evaluate the nutrients in detail, as shown by dishes scoring high in NRF 6.3, but low in the ANPS. We may have under-evaluated the nutritional value (density) of the dishes because of the limited number of nutrients used in the ANPS. However, we wanted to develop a system using the minimum number of necessary nutrients. Therefore, the advantages of ANPS are that it is simple to manage and easy to calculate for future scenarios. The elements and nutrients that were not included in the ANPS but are widely used in many popular NP models are energy, fiber, and added sugar (2, 3, 8). As for energy, unlike in Western countries, this element is not excessive in the Japanese population and was not included in evaluations. Since the ANPS is based on dish subcategories and serving size, most of the dishes were controlled by calorie content, except for bigger dishes such as dish subcategories 4, 5, 7, and 8. The largest calorie dish had 1,150 kcal (fried noodles topped with starchy sauce), and only 2.3% (25 of 1,089) of the dishes exceeded 660 kcal, which is 33% DV. In addition, these dishes tended to score low, with a median of 58.8, and low median sodium and SFA scores (3.0 and 2.5 points, respectively) that can compensate for the excessive energy of the dishes. For fiber, data from the National Health and Nutrition Survey in Japan showed that its main source was vegetable consumption in Japanese diets (22). Here, vegetables were included as a proxy indicator of fiber; therefore, this nutrient was excluded from the ANPS. As for added sugars, the amount in each dish category was mostly zero (Supplementary Table 1), we did not incorporate these into the evaluated nutrients. However, alterations of this should be considered in different cultural settings.

Finally, further validations are required to validate the usage of ANPS. In this report, we only conducted one type of validity testing, and so further tests that are needed include discriminant, construct, criterion, and predictive validity, ensuring the accuracy and usefulness of the ANPS (59, 60). In the future, we aim to first use this system for developing our in-house recipes and seasoning products, with the goal of producing nutritional dishes and products for the benefit of our customers. Overtime, we intend to expand this system by involving the food industry and authorities.

## Conclusion

A novel NP model concept ANPS was developed, which could be used to evaluate cooked dishes per serving size in Japan. This NP model has high sensitivity to sodium content of dishes, which is a major nutritional issue in Japan. Further research is still necessary to evaluate the accuracy and usefulness of ANPS as a system that could lead to a change in the eating behavior of wider populations.

## Data Availability Statement

The datasets presented in this article are not readily available because they are obtained from Excel Eiyo-kun ver.8 (Kenpakusha, Tokyo, Japan) and is following to the licenses/restriction. Requests to access the datasets should be directed to (tel:+81-03-3944-22611, website: https://www.kenpakusha.co.jp/).

## Author Contributions

CF, LC, AD, and YO contributed to the design of this research. CF and HJ analyzed the data and wrote the manuscript. All authors approved the final manuscript.

## Conflict of Interest

CF, HJ, LC, and YO were full-time employees of Ajinomoto Co., Inc., at the time of research. AD was the original founder of the Nutrient Rich Food Index, and has received grants, contracts, and honoraria from entities both public and private with an interest in nutrient profiling models. AD was served as a consultant to Ajinomoto Co., Inc., for this project.

## Publisher’s Note

All claims expressed in this article are solely those of the authors and do not necessarily represent those of their affiliated organizations, or those of the publisher, the editors and the reviewers. Any product that may be evaluated in this article, or claim that may be made by its manufacturer, is not guaranteed or endorsed by the publisher.

## Footnotes

1
https://park.ajinomoto.co.jp/recipe/
