# Supplementary material for: Nutrient Profiling of Japanese Dishes: The Development of a Novel Ajinomoto Group Nutrient Profiling System
Source: Front Nutr. 2022 Jul 29;9:912148. doi: 10.3389/fnut.2022.912148 (PMC9372512; doi:10.3389/fnut.2022.912148)
Supplement: Supplementary file 1 [file Data_Sheet_1.docx]

Supplementary Material

## Supplementary Table 1. Baseline data of nutrients in each dish category used for developing the Ajinomoto Group Nutrient Profiling System (ANPS) nutrient targets per dish category.

| Major dish group | Subcategory Number | Number of dishes | Energy (kcal) | | | | | | Protein (g) | | | | | | Fat (g) | | | | | |
| --- | --- | --- | --- | --- | --- | --- | --- | --- | --- | --- | --- | --- | --- | --- | --- | --- | --- | --- | --- | --- |
|  |  |  | Mean | SD | Median | IQR | | | Mean | SD | Median | IQR | | | Mean | SD | Median | IQR | | |
| Staple dish | 1 | 26 | 170.7 | 51.2 | 160.3 | 144.5 | - | 185.8 | 4.1 | 1.1 | 4.4 | 3.2 | - | 4.7 | 2.3 | 2.6 | 1.0 | 0.4 | - | 3.6 |
|  | 2 | 61 | 262.6 | 100.9 | 276.4 | 152.6 | - | 360.4 | 11.3 | 5.3 | 9.5 | 8.0 | - | 12.4 | 7.0 | 5.8 | 4.8 | 1.8 | - | 11.3 |
|  | 3 | 17 | 311.1 | 45.0 | 307.0 | 290.1 | - | 330.2 | 11.2 | 3.3 | 10.6 | 9.2 | - | 13.1 | 2.7 | 1.8 | 2.1 | 1.2 | - | 3.5 |
|  | 4 | 92 | 547.5 | 134.3 | 513.7 | 450.8 | - | 618.5 | 19.1 | 6.3 | 18.5 | 15.9 | - | 21.4 | 17.3 | 12.7 | 15.9 | 8.5 | - | 22.1 |
|  | 5 | 25 | 476.0 | 60.9 | 456.3 | 432.5 | - | 502.0 | 21.8 | 5.3 | 21.1 | 17.5 | - | 25.7 | 10.6 | 4.0 | 10.4 | 8.8 | - | 12.3 |
| Main dish | 6 | 90 | 243.1 | 110.2 | 232.9 | 161.9 | - | 299.4 | 15.3 | 4.9 | 15.0 | 11.8 | - | 18.0 | 13.4 | 8.8 | 12.3 | 6.4 | - | 18.6 |
|  | 7 | 69 | 272.3 | 95.4 | 271.6 | 199.1 | - | 329.2 | 14.6 | 6.0 | 13.7 | 9.3 | - | 19.3 | 14.7 | 8.4 | 13.4 | 9.1 | - | 19.1 |
|  | 8 | 17 | 399.6 | 150.6 | 380.9 | 329.7 | - | 427.0 | 21.5 | 6.0 | 20.7 | 18.1 | - | 24.0 | 24.2 | 15.5 | 18.9 | 14.6 | - | 30.4 |
| Soup | 9 | 34 | 67.8 | 65.2 | 46.5 | 26.9 | - | 65.2 | 3.3 | 1.5 | 3.1 | 2.3 | - | 4.3 | 3.1 | 4.5 | 0.8 | 0.2 | - | 3.1 |
|  | 10 | 11 | 141.8 | 70.8 | 143.8 | 77.5 | - | 202.3 | 4.2 | 1.2 | 4.7 | 2.9 | - | 5.6 | 6.7 | 5.0 | 8.5 | 1.5 | - | 10.7 |
|  | 11 | 46 | 155.4 | 72.8 | 131.8 | 105.1 | - | 212.9 | 10.0 | 3.8 | 9.1 | 7.4 | - | 11.7 | 6.7 | 5.0 | 5.7 | 3.3 | - | 8.2 |
| Side dish | 12 | 148 | 134.6 | 74.9 | 123.8 | 77.7 | - | 177.8 | 7.7 | 5.6 | 6.4 | 2.9 | - | 11.8 | 7.2 | 6.2 | 6.2 | 1.4 | - | 11.5 |
|  | 13 | 25 | 47.6 | 24.0 | 51.2 | 30.2 | - | 64.6 | 2.0 | 1.4 | 1.6 | 1.2 | - | 2.9 | 1.6 | 1.7 | 0.9 | 0.2 | - | 3.1 |
|  |  |  |  |  |  |  |  |  |  |  |  |  |  |  |  |  |  |  |  |  |

| Major dish group | Subcategory Number | Number of dishes | Saturated fat (g) | | | | | | Carbohydrate (g) | | | | | | Fiber (g) | | | | | |
| --- | --- | --- | --- | --- | --- | --- | --- | --- | --- | --- | --- | --- | --- | --- | --- | --- | --- | --- | --- | --- |
|  |  |  | Mean | SD | Median | IQR | | | Mean | SD | Median | IQR | | | Mean | SD | Median | IQR | | |
| Staple dish | 1 | 26 | 0.7 | 1.1 | 0.2 | 0.1 | - | 0.7 | 32.4 | 12.7 | 34.0 | 22.4 | - | 34.8 | 0.8 | 0.3 | 0.7 | 0.7 | - | 1.0 |
|  | 2 | 61 | 2.3 | 2.6 | 1.2 | 0.3 | - | 3.8 | 36.3 | 19.3 | 29.3 | 18.7 | - | 51.3 | 1.2 | 1.0 | 1.1 | 0.1 | - | 2.0 |
|  | 3 | 17 | 0.6 | 0.5 | 0.4 | 0.3 | - | 0.7 | 57.6 | 10.1 | 57.3 | 54.0 | - | 59.3 | 3.3 | 1.6 | 3.5 | 1.9 | - | 4.6 |
|  | 4 | 92 | 5.9 | 5.3 | 4.4 | 2.1 | - | 8.0 | 74.8 | 20.3 | 77.9 | 62.6 | - | 87.4 | 3.1 | 1.7 | 2.8 | 1.8 | - | 4.3 |
|  | 5 | 25 | 2.5 | 1.4 | 2.1 | 1.5 | - | 3.1 | 69.3 | 7.0 | 70.1 | 64.7 | - | 75.3 | 4.1 | 1.1 | 4.2 | 3.5 | - | 4.9 |
| Main dish | 6 | 90 | 3.4 | 2.8 | 2.5 | 1.4 | - | 5.1 | 13.2 | 11.4 | 10.4 | 5.1 | - | 16.7 | 1.3 | 1.6 | 0.9 | 0.5 | - | 1.6 |
|  | 7 | 69 | 4.0 | 3.1 | 3.0 | 1.6 | - | 5.1 | 18.8 | 9.5 | 17.1 | 11.9 | - | 24.9 | 2.8 | 1.5 | 2.5 | 1.6 | - | 3.3 |
|  | 8 | 17 | 8.0 | 5.1 | 7.2 | 4.4 | - | 10.6 | 21.6 | 8.5 | 22.0 | 14.3 | - | 27.6 | 4.9 | 3.5 | 4.2 | 3.4 | - | 5.4 |
| Soup | 9 | 34 | 1.3 | 2.5 | 0.1 | 0.1 | - | 0.5 | 7.1 | 6.9 | 3.8 | 2.4 | - | 10.2 | 1.1 | 1.0 | 0.8 | 0.5 | - | 1.3 |
|  | 10 | 11 | 3.2 | 3.1 | 1.4 | 0.2 | - | 6.4 | 16.3 | 6.8 | 16.1 | 10.9 | - | 20.2 | 2.1 | 0.9 | 2.0 | 1.6 | - | 2.2 |
|  | 11 | 46 | 2.4 | 2.6 | 1.5 | 0.7 | - | 2.8 | 12.9 | 12.9 | 8.9 | 4.2 | - | 14.5 | 1.8 | 1.5 | 1.5 | 0.8 | - | 2.3 |
| Side dish | 12 | 148 | 1.6 | 1.9 | 1.0 | 0.3 | - | 2.2 | 9.2 | 8.3 | 6.5 | 3.5 | - | 12.6 | 1.6 | 1.8 | 1.0 | 0.4 | - | 2.0 |
|  | 13 | 25 | 0.3 | 0.4 | 0.2 | 0.0 | - | 0.5 | 6.4 | 3.9 | 6.5 | 3.8 | - | 9.1 | 1.3 | 1.0 | 1.1 | 0.6 | - | 1.9 |
|  |  |  |  |  |  |  |  |  |  |  |  |  |  |  |  |  |  |  |  |  |

| Major dish group | Subcategory Number | Number of dishes | Sodium (mg) | | | | | | Potassium (mg) | | | | | | Calcium (mg) | | | | | |
| --- | --- | --- | --- | --- | --- | --- | --- | --- | --- | --- | --- | --- | --- | --- | --- | --- | --- | --- | --- | --- |
|  |  |  | Mean | SD | Median | IQR | | | Mean | SD | Median | IQR | | | Mean | SD | Median | IQR | | |
| Staple dish | 1 | 26 | 236.2 | 178.9 | 211.9 | 105.1 | - | 334.3 | 89.1 | 26.5 | 87.5 | 67.0 | - | 113.1 | 16.5 | 17.3 | 8.0 | 6.0 | - | 18.1 |
|  | 2 | 61 | 537.6 | 406.0 | 407.4 | 237.7 | - | 758.0 | 204.5 | 110.2 | 165.8 | 133.3 | - | 260.4 | 47.3 | 62.4 | 26.7 | 13.7 | - | 41.3 |
|  | 3 | 17 | 1268.0 | 340.5 | 1259.4 | 1188.3 | - | 1498.8 | 326.6 | 164.4 | 260.4 | 207.3 | - | 364.1 | 43.7 | 18.0 | 36.7 | 32.0 | - | 50.4 |
|  | 4 | 92 | 1265.1 | 815.7 | 1044.1 | 849.6 | - | 1423.0 | 414.0 | 212.8 | 362.9 | 266.5 | - | 495.9 | 94.4 | 111.9 | 59.0 | 44.9 | - | 86.2 |
|  | 5 | 25 | 1557.6 | 457.9 | 1517.7 | 1293.9 | - | 1636.8 | 581.1 | 213.3 | 510.0 | 416.1 | - | 675.5 | 84.8 | 26.5 | 78.6 | 63.4 | - | 94.0 |
| Main dish | 6 | 90 | 684.4 | 243.9 | 649.2 | 504.3 | - | 806.2 | 349.0 | 122.8 | 345.3 | 269.6 | - | 408.5 | 71.9 | 67.8 | 49.7 | 24.6 | - | 103.1 |
|  | 7 | 69 | 733.4 | 348.7 | 746.1 | 482.5 | - | 919.5 | 544.2 | 206.8 | 486.0 | 385.8 | - | 633.2 | 99.5 | 120.7 | 44.8 | 34.5 | - | 120.6 |
|  | 8 | 17 | 1064.7 | 369.2 | 1049.3 | 854.5 | - | 1351.0 | 932.7 | 283.4 | 914.3 | 726.5 | - | 1117.9 | 142.5 | 88.9 | 110.2 | 91.8 | - | 167.0 |
| Soup | 9 | 34 | 606.9 | 127.6 | 566.5 | 537.6 | - | 691.9 | 290.3 | 176.1 | 244.6 | 199.1 | - | 306.6 | 37.6 | 35.6 | 26.0 | 15.4 | - | 41.3 |
|  | 10 | 11 | 754.2 | 169.8 | 710.5 | 654.3 | - | 753.9 | 372.4 | 103.5 | 323.8 | 313.6 | - | 426.6 | 57.9 | 39.2 | 36.7 | 30.5 | - | 96.3 |
|  | 11 | 46 | 837.6 | 305.2 | 741.5 | 660.8 | - | 953.0 | 418.6 | 151.8 | 390.1 | 297.2 | - | 501.4 | 58.4 | 39.8 | 48.0 | 28.1 | - | 74.3 |
| Side dish | 12 | 148 | 452.1 | 258.7 | 407.7 | 298.6 | - | 565.4 | 286.0 | 189.3 | 229.3 | 175.1 | - | 338.2 | 44.1 | 38.4 | 30.6 | 18.1 | - | 53.8 |
|  | 13 | 25 | 397.4 | 424.1 | 234.8 | 27.0 | - | 677.4 | 168.7 | 152.9 | 124.0 | 83.7 | - | 203.3 | 22.7 | 20.9 | 14.8 | 7.8 | - | 32.3 |
|  |  |  |  |  |  |  |  |  |  |  |  |  |  |  |  |  |  |  |  |  |

| Major dish group | Subcategory Number | Number of dishes | Magnesium (mg) | | | | | | Iron (mg) | | | | | | VitaminA as  retinol activity equivalents (μg) | | | | | |
| --- | --- | --- | --- | --- | --- | --- | --- | --- | --- | --- | --- | --- | --- | --- | --- | --- | --- | --- | --- | --- |
|  |  |  | Mean | SD | Median | IQR | | | Mean | SD | Median | IQR | | | Mean | SD | Median | IQR | | |
| Staple dish | 1 | 26 | 13.3 | 3.3 | 12.9 | 11.8 | - | 14.0 | 0.4 | 0.1 | 0.3 | 0.3 | - | 0.4 | 36.7 | 25.5 | 42.2 | 8.1 | - | 55.0 |
|  | 2 | 61 | 27.4 | 15.7 | 24.6 | 17.3 | - | 32.2 | 0.9 | 0.8 | 0.7 | 0.5 | - | 1.0 | 51.1 | 84.4 | 30.9 | 5.2 | - | 62.0 |
|  | 3 | 17 | 56.7 | 25.0 | 60.5 | 33.7 | - | 75.7 | 1.3 | 0.7 | 0.8 | 0.7 | - | 1.9 | 18.7 | 19.1 | 8.1 | 4.9 | - | 36.1 |
|  | 4 | 92 | 52.6 | 24.2 | 45.4 | 35.5 | - | 60.5 | 1.7 | 0.9 | 1.6 | 1.1 | - | 2.2 | 120.4 | 144.5 | 94.4 | 51.3 | - | 147.0 |
|  | 5 | 25 | 69.5 | 23.2 | 65.9 | 53.4 | - | 89.4 | 2.1 | 0.8 | 1.9 | 1.6 | - | 2.4 | 73.2 | 65.4 | 52.1 | 8.5 | - | 114.0 |
| Main dish | 6 | 90 | 52.9 | 49.2 | 34.4 | 26.7 | - | 51.5 | 1.3 | 0.7 | 1.2 | 0.8 | - | 1.8 | 68.9 | 133.7 | 33.5 | 10.6 | - | 78.7 |
|  | 7 | 69 | 56.2 | 50.1 | 40.2 | 29.4 | - | 59.3 | 1.7 | 1.1 | 1.3 | 0.9 | - | 2.1 | 116.0 | 130.6 | 88.0 | 24.5 | - | 158.5 |
|  | 8 | 17 | 76.6 | 46.1 | 59.5 | 43.3 | - | 83.0 | 2.5 | 1.2 | 2.4 | 1.5 | - | 3.1 | 213.8 | 122.3 | 209.0 | 89.5 | - | 273.2 |
| Soup | 9 | 34 | 28.0 | 19.7 | 23.4 | 16.8 | - | 31.5 | 0.5 | 0.4 | 0.5 | 0.3 | - | 0.7 | 36.5 | 53.7 | 13.2 | 3.6 | - | 46.2 |
|  | 10 | 11 | 30.4 | 13.2 | 25.9 | 24.4 | - | 30.2 | 0.6 | 0.1 | 0.6 | 0.5 | - | 0.7 | 126.0 | 123.2 | 85.1 | 41.4 | - | 186.9 |
|  | 11 | 46 | 38.2 | 23.1 | 31.3 | 25.5 | - | 38.7 | 1.0 | 0.5 | 0.9 | 0.5 | - | 1.4 | 97.1 | 211.7 | 44.5 | 16.8 | - | 89.3 |
| Side dish | 12 | 148 | 29.8 | 21.9 | 25.9 | 15.8 | - | 34.6 | 0.9 | 0.7 | 0.7 | 0.4 | - | 1.1 | 128.8 | 699.0 | 30.4 | 11.2 | - | 88.5 |
|  | 13 | 25 | 17.8 | 18.7 | 11.6 | 6.9 | - | 16.1 | 0.6 | 0.9 | 0.3 | 0.2 | - | 0.5 | 22.7 | 63.9 | 3.2 | 0.0 | - | 12.1 |
|  |  |  |  |  |  |  |  |  |  |  |  |  |  |  |  |  |  |  |  |  |

| Major dish group | Subcategory Number | Number of dishes | Vitamin D (µg) | | | | | | Vitamin B1 (mg) | | | | | | Vitamin B2 (mg) | | | | | |
| --- | --- | --- | --- | --- | --- | --- | --- | --- | --- | --- | --- | --- | --- | --- | --- | --- | --- | --- | --- | --- |
|  |  |  | Mean | SD | Median | IQR | | | Mean | SD | Median | IQR | | | Mean | SD | Median | IQR | | |
| Staple dish | 1 | 26 | 0.2 | 0.5 | 0.0 | 0.0 | - | 0.3 | 0.0 | 0.0 | 0.0 | 0.0 | - | 0.0 | 0.1 | 0.0 | 0.1 | 0.0 | - | 0.1 |
|  | 2 | 61 | 1.2 | 2.5 | 0.2 | 0.0 | - | 0.8 | 0.1 | 0.1 | 0.1 | 0.1 | - | 0.1 | 0.1 | 0.1 | 0.1 | 0.1 | - | 0.1 |
|  | 3 | 17 | 0.3 | 0.3 | 0.3 | 0.0 | - | 0.4 | 0.1 | 0.1 | 0.1 | 0.1 | - | 0.1 | 0.1 | 0.1 | 0.1 | 0.1 | - | 0.1 |
|  | 4 | 92 | 1.3 | 4.0 | 0.3 | 0.2 | - | 0.9 | 0.2 | 0.2 | 0.2 | 0.1 | - | 0.3 | 0.2 | 0.2 | 0.2 | 0.1 | - | 0.3 |
|  | 5 | 25 | 1.2 | 3.9 | 0.3 | 0.1 | - | 0.7 | 0.3 | 0.3 | 0.2 | 0.1 | - | 0.5 | 0.2 | 0.1 | 0.2 | 0.2 | - | 0.3 |
| Main dish | 6 | 90 | 1.9 | 4.5 | 0.3 | 0.1 | - | 1.0 | 0.1 | 0.1 | 0.1 | 0.1 | - | 0.2 | 0.2 | 0.3 | 0.2 | 0.1 | - | 0.2 |
|  | 7 | 69 | 1.6 | 4.7 | 0.3 | 0.1 | - | 0.6 | 0.2 | 0.2 | 0.1 | 0.1 | - | 0.3 | 0.2 | 0.3 | 0.2 | 0.1 | - | 0.3 |
|  | 8 | 17 | 1.0 | 2.5 | 0.4 | 0.3 | - | 0.5 | 0.3 | 0.2 | 0.2 | 0.2 | - | 0.3 | 0.3 | 0.1 | 0.3 | 0.3 | - | 0.4 |
| Soup | 9 | 34 | 0.1 | 0.1 | 0.0 | 0.0 | - | 0.2 | 0.1 | 0.1 | 0.1 | 0.0 | - | 0.1 | 0.1 | 0.1 | 0.1 | 0.0 | - | 0.1 |
|  | 10 | 11 | 0.2 | 0.2 | 0.1 | 0.0 | - | 0.3 | 0.1 | 0.1 | 0.1 | 0.1 | - | 0.1 | 0.1 | 0.1 | 0.1 | 0.1 | - | 0.2 |
|  | 11 | 46 | 1.2 | 2.4 | 0.3 | 0.0 | - | 0.7 | 0.2 | 0.1 | 0.1 | 0.1 | - | 0.2 | 0.2 | 0.1 | 0.1 | 0.1 | - | 0.2 |
| Side dish | 12 | 148 | 0.8 | 2.4 | 0.1 | 0.0 | - | 0.4 | 0.1 | 0.1 | 0.1 | 0.0 | - | 0.1 | 0.1 | 0.1 | 0.1 | 0.0 | - | 0.2 |
|  | 13 | 25 | 0.3 | 1.0 | 0.0 | 0.0 | - | 0.0 | 0.0 | 0.0 | 0.0 | 0.0 | - | 0.0 | 0.0 | 0.0 | 0.0 | 0.0 | - | 0.0 |
|  |  |  |  |  |  |  |  |  |  |  |  |  |  |  |  |  |  |  |  |  |

| Major dish group | Subcategory Number | Number of dishes | Vitamin C (mg) | | | | | | Vegetables (g) | | | | | | Added sugar (g) | | | | | |
| --- | --- | --- | --- | --- | --- | --- | --- | --- | --- | --- | --- | --- | --- | --- | --- | --- | --- | --- | --- | --- |
|  |  |  | Mean | SD | Median | IQR | | | Mean | SD | Median | IQR | | | Mean | SD | Median | IQR | | |
| Staple dish | 1 | 26 | 2.5 | 2.0 | 2.4 | 0.8 | - | 3.6 | 6.5 | 10.6 | 0.0 | 0.0 | - | 9.5 | 0.7 | 2.5 | 0.0 | 0.0 | - | 0.5 |
|  | 2 | 61 | 3.8 | 5.9 | 1.6 | 0.4 | - | 3.5 | 10.4 | 15.5 | 1.0 | 0.0 | - | 15.0 | 1.0 | 2.3 | 1.0 | 0.0 | - | 1.0 |
|  | 3 | 17 | 3.7 | 3.5 | 2.3 | 1.0 | - | 6.1 | 12.9 | 13.7 | 7.0 | 3.0 | - | 20.0 | 0.1 | 0.4 | 0.0 | 0.0 | - | 0.0 |
|  | 4 | 92 | 10.5 | 11.5 | 6.0 | 3.1 | - | 14.0 | 40.6 | 45.1 | 30.3 | 7.0 | - | 51.1 | 1.5 | 3.0 | 0.0 | 0.0 | - | 1.0 |
|  | 5 | 25 | 8.1 | 6.1 | 6.6 | 4.2 | - | 12.9 | 35.0 | 28.3 | 31.0 | 5.0 | - | 55.0 | 1.0 | 1.9 | 0.0 | 0.0 | - | 0.5 |
| Main dish | 6 | 90 | 7.0 | 9.6 | 3.5 | 1.2 | - | 7.6 | 19.8 | 16.4 | 20.5 | 2.3 | - | 34.3 | 1.9 | 2.4 | 1.0 | 0.0 | - | 3.0 |
|  | 7 | 69 | 24.9 | 20.1 | 18.0 | 7.8 | - | 36.9 | 84.2 | 33.4 | 71.1 | 57.5 | - | 101.0 | 2.2 | 3.2 | 1.0 | 0.0 | - | 3.0 |
|  | 8 | 17 | 41.8 | 26.0 | 32.8 | 22.7 | - | 54.5 | 160.3 | 64.8 | 150.0 | 109.9 | - | 180.0 | 1.0 | 2.6 | 0.0 | 0.0 | - | 0.0 |
| Soup | 9 | 34 | 5.7 | 8.6 | 1.6 | 0.7 | - | 4.5 | 13.3 | 12.8 | 5.0 | 3.0 | - | 25.0 | 0.0 | 0.0 | 0.0 | 0.0 | - | 0.0 |
|  | 10 | 11 | 13.8 | 11.3 | 5.3 | 4.1 | - | 23.6 | 78.5 | 34.4 | 60.0 | 56.5 | - | 85.3 | 0.1 | 0.4 | 0.0 | 0.0 | - | 0.0 |
|  | 11 | 46 | 5.8 | 5.4 | 4.3 | 1.6 | - | 7.9 | 32.3 | 30.2 | 21.5 | 9.3 | - | 44.3 | 0.0 | 0.2 | 0.0 | 0.0 | - | 0.0 |
| Side dish | 12 | 148 | 10.2 | 11.8 | 5.9 | 2.0 | - | 13.2 | 39.0 | 36.9 | 34.0 | 3.0 | - | 60.0 | 1.8 | 2.9 | 0.0 | 0.0 | - | 3.0 |
|  | 13 | 25 | 5.8 | 8.9 | 2.4 | 0.0 | - | 5.4 | 12.4 | 16.2 | 0.0 | 0.0 | - | 25.0 | 0.9 | 1.5 | 0.0 | 0.0 | - | 1.5 |

1 Values are expressed as the mean, standard deviation (SD), median, and interquartile range (IQR).

2 Nutritional values were calculated from the 661 recipes stored in the nutritional calculation software, Excel Eiyo-kun ver.8 (Kenpakusha, Tokyo, Japan).

**Supplementary Table 2.** Nutrient standards for the nutrient-rich food index (NRF) 6.3 (calculation based on the Ajinomoto Group Nutrient Profiling System (ANPS) ANPS and Food and Drug Administration values).

| Nutrient | Amount |
| --- | --- |
| Protein | 66 g |
| *Fiber | 28 g |
|  |  |
| *Vitamin D | 20 mcg |
| *Calcium | 1300 mg |
| *Iron | 18 mg |
| *Potassium | 4700 mg |
|  |  |
| Saturated fatty acids | 22.2 g |
| *Added sugar | 50 g |
| Sodium | 2756 mg |

*Nutrient standards adapted from the Food and Drug Administration values. Nutrients without symbols are adapted from the ANPS.

## Supplementary Table 3. The Ajinomoto Group Nutrient Profiling System (ANPS) score of each dish category for the 1089 dishes evaluated.

| Major dish group | Subcategory Number | Number of dishes | ANPS score | | | | | |
| --- | --- | --- | --- | --- | --- | --- | --- | --- |
|  |  |  | Mean | SD | Median | IQR | | |
| Staple dish | 1 | 30 | 66.2 | 13.4 | 69.4 | 59.1 | - | 75.0 |
|  | 2 | 78 | 65.7 | 18.0 | 70.0 | 51.9 | - | 75.0 |
|  | 3 | 21 | 60.4 | 15.9 | 56.3 | 50.0 | - | 71.9 |
|  | 4 | 141 | 73.1 | 15.8 | 71.3 | 60.7 | - | 87.5 |
|  | 5 | 29 | 60.3 | 12.5 | 60.0 | 51.3 | - | 72.5 |
| Main dish | 6 | 138 | 66.9 | 16.9 | 66.3 | 54.7 | - | 80.0 |
|  | 7 | 139 | 70.5 | 14.6 | 71.3 | 62.5 | - | 80.0 |
|  | 8 | 51 | 77.4 | 12.5 | 77.5 | 70.0 | - | 87.5 |
| Soup | 9 | 68 | 71.4 | 16.5 | 71.9 | 56.9 | - | 85.0 |
|  | 10 | 60 | 78.5 | 13.8 | 80.5 | 70.0 | - | 88.5 |
|  | 11 | 62 | 52.1 | 13.3 | 51.9 | 43.5 | - | 61.3 |
| Side dish | 12 | 235 | 63.7 | 15.1 | 67.5 | 52.5 | - | 75.0 |
|  | 13 | 37 | 70.0 | 18.0 | 71.3 | 55.0 | - | 85.0 |
| Total dish |  | 1089 | 67.7 | 16.5 | 70.0 | 55.0 | - | 78.8 |

1 Values are expressed as the mean, standard deviation (SD), median, and interquartile range (IQR).

## Supplementary Table 4. The Ajinomoto Group Nutrient Profiling System (ANPS) nutritional score of each dish category for the 1089 dishes evaluated.

| Major dish group | Subcategory Number | Number of dishes | Protein score | | | | | | Vegetable score | | | | | | SFA score | | | | | | | Sodium score | | | | | | | | | |
| --- | --- | --- | --- | --- | --- | --- | --- | --- | --- | --- | --- | --- | --- | --- | --- | --- | --- | --- | --- | --- | --- | --- | --- | --- | --- | --- | --- | --- | --- | --- | --- |
|  |  |  | Mean | SD | Median | IQR | | | Mean | SD | Median | IQR | | | Mean | SD | Median | IQR | | | | Mean | | SD | | Median | | IQR | | |  |
| Staple dish | 1 | 30 | 9.1 | 1.7 | 10.0 | 8.8 | - | 10.0 | 2.2 | 3.5 | 0.0 | 0.0 | - | 4.3 | 9.0 | 3.1 | 10.0 | 10.0 | - | 10.0 | 6.2 | | 3.8 | | 7.3 | | 2.5 | | - | 10.0 |  |
|  | 2 | 78 | 8.7 | 1.5 | 9.0 | 8.0 | - | 10.0 | 4.2 | 4.5 | 2.0 | 0.0 | - | 10.0 | 7.4 | 4.2 | 10.0 | 3.0 | - | 10.0 | 5.9 | | 4.1 | | 7.0 | | 1.4 | | - | 10.0 |  |
|  | 3 | 21 | 9.1 | 1.5 | 10.0 | 8.5 | - | 10.0 | 5.3 | 4.5 | 5.0 | 1.0 | - | 10.0 | 7.8 | 4.0 | 10.0 | 6.5 | - | 10.0 | 2.0 | | 3.1 | | 0.0 | | 0.0 | | - | 3.8 |  |
|  | 4 | 141 | 9.1 | 1.6 | 10.0 | 9.0 | - | 10.0 | 5.7 | 3.9 | 6.0 | 2.0 | - | 10.0 | 7.7 | 3.4 | 10.0 | 5.5 | - | 10.0 | 6.7 | | 3.3 | | 7.5 | | 5.0 | | - | 9.5 |  |
|  | 5 | 29 | 9.3 | 1.1 | 10.0 | 8.5 | - | 10.0 | 4.7 | 4.0 | 5.0 | 0.0 | - | 9.0 | 9.2 | 2.3 | 10.0 | 10.0 | - | 10.0 | 0.9 | | 2.2 | | 0.0 | | 0.0 | | - | 1.0 |  |
| Main dish | 6 | 138 | 8.8 | 1.8 | 10.0 | 7.8 | - | 10.0 | 6.1 | 4.4 | 9.5 | 0.0 | - | 10.0 | 7.8 | 3.7 | 10.0 | 7.0 | - | 10.0 | 4.1 | | 3.4 | | 4.0 | | 0.9 | | - | 7.0 |  |
|  | 7 | 139 | 8.5 | 2.0 | 10.0 | 7.0 | - | 10.0 | 8.4 | 1.8 | 9.0 | 7.0 | - | 10.0 | 7.2 | 4.0 | 10.0 | 5.0 | - | 10.0 | 4.1 | | 3.8 | | 4.0 | | 0.0 | | - | 7.5 |  |
|  | 8 | 51 | 8.5 | 2.1 | 10.0 | 7.0 | - | 10.0 | 9.0 | 1.9 | 10.0 | 9.0 | - | 10.0 | 6.6 | 4.2 | 10.0 | 3.0 | - | 10.0 | 6.8 | | 3.1 | | 7.5 | | 4.5 | | - | 9.5 |  |
| Soup | 9 | 68 | 7.6 | 2.9 | 9.0 | 5.0 | - | 10.0 | 6.9 | 4.1 | 10.0 | 2.0 | - | 10.0 | 8.5 | 3.4 | 10.0 | 10.0 | - | 10.0 | 5.5 | | 3.3 | | 5.0 | | 2.5 | | - | 9.0 |  |
|  | 10 | 60 | 8.2 | 2.7 | 10.0 | 6.3 | - | 10.0 | 9.2 | 1.2 | 10.0 | 8.0 | - | 10.0 | 7.8 | 3.6 | 10.0 | 5.0 | - | 10.0 | 6.2 | | 3.7 | | 7.8 | | 2.6 | | - | 9.5 |  |
|  | 11 | 62 | 8.5 | 1.6 | 9.0 | 7.0 | - | 10.0 | 5.2 | 4.1 | 5.5 | 1.0 | - | 10.0 | 5.1 | 4.6 | 5.5 | 0.0 | - | 10.0 | 2.0 | | 2.6 | | 0.8 | | 0.0 | | - | 3.6 |  |
| Side dish | 12 | 235 | 4.9 | 3.6 | 4.0 | 2.0 | - | 9.0 | 6.5 | 4.1 | 9.0 | 2.0 | - | 10.0 | 6.9 | 4.2 | 10.0 | 3.0 | - | 10.0 | 7.1 | | 3.2 | | 8.0 | | 5.5 | | - | 10.0 |  |
|  | 13 | 37 | 5.1 | 3.5 | 4.0 | 2.5 | - | 8.5 | 5.4 | 4.7 | 7.0 | 0.0 | - | 10.0 | 9.8 | 1.5 | 10.0 | 10.0 | - | 10.0 | 7.7 | | 3.5 | | 10.0 | | 4.5 | | - | 10.0 |  |

1 Values are expressed as the mean, standard deviation (SD), median, and interquartile range (IQR).

## Supplementary Table 5. Pearson’s correlation coefficients among the Ajinomoto Group Nutrient Profiling System (ANPS) and respective nutrient-rich food index (NRF) 6.3 nutrient scores

| Nutrients | r | *p-*value |
| --- | --- | --- |
| Protein | 0.0138 | 0.6482 |
| **Fiber** | **0.1203** | **<0.001** |
| Calcium | -0.0494 | 0.1036 |
| Iron | 0.0204 | 0.5005 |
| Vitamin D | 0.0565 | 0.0623 |
| **Potassium** | **0.1636** | **<0.001** |
| **Saturated fatty acid** | **-0.2723** | **<0.001** |
| **Sodium** | **-0.3244** | **<0.001** |
| **Added sugar** | **-0.1031** | **<0.001** |

Significant correlations are highlighted in bold.
